# Ultrarapid Opioid Detoxification: Current Status in Iran and Controversies

**DOI:** 10.5812/ijhrba.13140

**Published:** 2013-12-12

**Authors:** Seyyed Mojtaba Yassini Ardekani, Sara Yassini Ardekani

**Affiliations:** 1 Department of Psychiatry, Yazd Shahid Sadoughi University of Medical Sciences, Yazd, IR Iran; 2 Department of Medicine, Yazd Shahid Sadoughi University of Medical Sciences, Yazd, IR Iran

**Keywords:** Opioid-Related Disorders, Therapeutics, Methadone Detoxification, Drug

## 1. Introduction

Opioid addiction is a multifactorial problem involving physiological, psychological, genetic, behavioral and environmental factors. No single treatment approach is effective in all cases. Traditional methods of treatment include tapering with methadone or buprenorphine or discontinuing opioids and administering oral clonidine to ameliorate withdrawal syndrome. Even when pharmacologic agents are used in the management of opioid withdrawal, there is often a significant amount of patient discomfort (1).

Opiates detoxification can be accomplished on an inpatient or outpatient basis. Withdrawal symptoms usually last 72 hours or less regardless of the agent used for detoxification. Addicts may complain of residual withdrawal symptoms for days or even weeks. After detoxification, maintenance therapy is of great importance in abstinence period. Many clinicians recommend daily administration of an orally active opiate blocking agent (naltrexone, ReVia) (2).

## 2. Rapid and Ultra-rapid Detoxification

Attempts have been made to induce and shorten the opiate withdrawal by clonidine and opiate antagonists since 1970s (3) Blachley et al. (4) were one of the first groups suggested the use of anesthesia to make the process of detoxification more humane. For the first time the ultrarapid detoxification method developed by Lomier et al. (5), based on earlier rapid detoxification methods published by researchers at the Yale University (6, 7). Since that time there has been several modifications and improvements in the technique of ultrarapid opioid detoxification (8). The common underlying theme in all the programmers of UROD is to shorten the detoxification process to a 6-8 hour period by precipitating withdrawal following the administration of opioid antagonists under general anesthesia, blunting the awareness of physical discomfort by deep sedation or anesthesia appropriately and shortening the lag time between a patient’s last dose of opioid and his or her transfer (induction) on to naltrexone maintenance. Johnson and Carr (9) suggested that UROD is a procedure using general anesthesia of less than 6 hours, and ROD is a procedure with deep sedation of about 6-72 hours (8).

## 3. UROD in Iran

The history of UROD in Iran dates back to about 13 years ago after doing the first one in the capital city, Tehran, and rapidly distributed to other large cities, and then to all over the country. It was probably due to the high prevalence of opiate addiction, introducing this method as a brief and painless procedure in advertisements, and the most important was presumably the supposition of addicts that in this method the blood is exchanged and the chance of relapse is very low or not at all. Day by day the number of addicts requesting this kind of detoxification was increasing and in addition to the increasing number of involved psychiatrists, anesthesiologists started to manage addicts independently. After few years the Ministry of Health and Medical Sciences decided to limit this kind of detoxification to educational and research centers since 2007 due to mismanagement in some centers, and the belief that this method is not more effective than other methods. At the same time methadone detoxification was prohibited and buprenorphine detoxification was substituted in detoxification centers, and methadone maintenance therapy centers increased rapidly, so there was a shift of addicts from URD centers to buprenorphine detoxification and methadone maintenance therapy centers (10).

## 4. Available Evidences

Considering the design of studies, 9 UROD and 12 ROD and according to O'Cannor and Kosten (11) extensive research, most of studies used general anesthesia, and only three studies included control group. Most of the studies focused on the completion of detoxification or the severity of withdrawal of symptoms. In a clinical analytic outcome study, 153 addicts enrolled in URD and the severity of withdrawal symptoms was rated 12 hours after detoxification using SOWS. Results were indicated of well controlled withdrawal syndrome (12).

Considering the long-term efficacy of URD Seoane et al. (13) reported that 93% of patients were abstinent after one month. Rabinowitz reported that 57% of 113 patients who were detoxified by this method were in abstinence after 12 months; and in Brewer study, 76% of 510 addicts were in abstinence after 4 months (12). A literature review was performed by Bell et al. (14) from 1980-2000 and 21 studies on naltrexone-accelerated procedure were evaluated. According to this review withdrawal syndrome was quite protracted with a mean duration of 3-4 days. The range of follow up study varied from 3 months to one year (15), and the range of abstinence rate varied from 20% at 6 months (16) to 68% at 12 months (17).

Comparing this method to methadone-tapering method, abstinence rate is significantly higher in UROD (67%) than methadone group (33%) (17). Lawental (18) in a retrospective follow-up study, compared the abstinence rate after 12 and 18 months between subjects undergone UROD and those who had undergone a 30 day inpatient detoxification. He found that 22% in the former group were in abstinence compared to 42% in the latter.

Three months follow up study comparing abstinence rate and withdrawal effects of UROD with standard methadone tapering method shows significantly higher abstinence rate and milder withdrawal symptoms in UROD group at 1 and 2 months follow up, but it was not significant at 3 months (19). A follow up study of 16 patients undergone UROD showed that 14 of them relapsed after 30 months (20).

## 5. Controversies

To evaluate the usefulness of URD we have to consider its cons and pros. The procedure nature enforces patients to complete the process, so its efficacy is 100% in the short-term and greater number of patients enter long-term treatment using naltrexone maintenance and psychological support. Beside, severity of withdrawal syndrome is at minimum level comparing to conventional methods, so some patients who do not enter treatment because of the fear of the pain are motivated to accept substitute detoxification with UROD process (12). According to a school of thought it is the physician responsibility to provide convenience in any procedure (21).

Preferring UROD method in addicts may be due to the fear of developing severe withdrawal syndrome in traditional method, frustration in methadone detoxification and craving to abuse methadone more and more (22). As long as neonates and children are chemically but not psychologically dependent, UROD method could be effective for detoxification in this age group (23).

Talking about harms and pitfalls of UROD, we have to consider morbidity and mortality rates. There is a report about mortality rate of 4 of 10000 and reports about cases of suicidal commit, thyroid suppression, respiratory distress, and renal failure following UROD (24). One of the most important and critical accidental problems is abusing high dose of opiate following UROD with naltrexone resulting in poisoning and even death (25).

Naltrexone implant has its own consequences including pulmonary edema, aspiration pneumonia, protracted withdrawal syndrome and six deaths in one of therapeutic centers (26). Low education, joblessness and legal problems are factors with direct significant association with relapse in URD (27).

There is no clear evidence that this procedure, as opposed to the standard detoxification, leads to greater abstinence rates; although, the immediate and short-term outcomes are encouraging whether these can be considered as valid outcomes, regarding the procedure nature, is a debatable issue.

The American Society of Addiction Medicine (ASAM) has issued elaborate recommendations (28) for UROD incorporating many of the above ideas. It recommends that any method of opioid detoxification is only a first step, and is not an effective treatment of opioid addiction per se.

## 6. Author's Experiences

The author has personally performed more than 2500 UROD during the past 12 years and what is coming in following parts are the most important issues we noticed.

## 7. Clients

In a sample size of 153 individuals referred for UROD, 98% of addicts were male and 0.2% were female. Considering educational status, 20.3% had primary school, 30.7% middle school, 34% high school and 15% college level education. The mean age was 35.3 ranging from 20 to 62 years (12).

## 8. Procedure

Detoxification was performed under general anesthesia for about 4 hours. Induction of general anesthesia was performed by propofol and atracurium. Shortly after intubation and stabilization of patient, IV drip of naloxone was initiated for patient and maintained for 3 hours. Knowing about the half-life of naloxone, after discontinuation of naloxone IV drip, patient was received IV therapy for one hour. Cardiopulmonary status was monitored continuously; while patient was under general anesthesia. Two mg of ondansetron was administered subcutaneously to control diarrhea and vomiting (29).

## 9. Cardiovascular Changes During Procedure

Blood pressure of 36.6% of individuals was in the range of 40-140. 24.2% developed hypotension, 35.3% developed hypertension and in 3.9% blood pressure was variable. The heart rate in 75% of the cases was in the normal range (60-120), 11% developed tachycardia, 12.4% developed bradycardia, and in 7% the heart rate was variable (12).

## 10. Withdrawal Syndrome

According to the report of Yassini et al. (12), the severity of withdrawal symptoms, measuring by SWOS was fair in 35.9% of patients, good in 20.3% and excellent in the remaining, and generally was less sever in those who were polyopiate substance abuser.

## 11. Complications

In one study, among 25 individuals who underwent URD procedure, one who injected 32 buprenorphine in a day developed severe depression 58 hours after detoxification, two of them developed delirium and one of them developed pulmonary edema (30).

## 12. Follow Up

Follow up studies in the field of addiction in Iranian culture is not possible or at least is very difficult. As long as they suppose that their inpatient chart will make some difficulties, legal or familial, for them they usually give wrong name or contact number, which makes follow up impossible. But according to what I have heard from my clients in my office there are individuals who have underwent UROD 10 years ago and they are still in abstinence.

## 13. Conclusions

In any type of detoxification, it is per se is the first step of addiction management, and what is important for the clients is the duration and severity of withdrawal syndrome. Studies are indicative of less withdrawal syndrome severity and less duration, but as long as complication of URD is more than other detoxification methods, it is recommended to use this method for educated, young and healthy individuals with enough motivation and familial support. Generally its use in clinical settings is not supportable until a clearly positive risk-benefit relationship can be demonstrated. Further research on UROD should be conducted.
